# Changing incidence and management of penetrating neck injuries in the South East London trauma centre

**DOI:** 10.1308/003588412X13171221590052

**Published:** 2012-05

**Authors:** R Harris, C Olding, C Lacey, R Bentley, KM Schulte, D Lewis, N Kandasamy, R Oakley

**Affiliations:** ^1^Whipps Cross University Hospital NHS Foundation TrustUK; ^2^King’s College Hospital NHS Foundation TrustUK; ^3^Guy’s and St Thomas’ NHS Foundation TrustUK

**Keywords:** Wounds, Penetrating, Neck injuries, Stab, Gunshot, Treatment protocol

## Abstract

**INTRODUCTION:**

A total of 17 cases of penetrating neck injury were managed by the otolaryngology team at King’s College Hospital over a 3-year period in the 1980s. In April 2010 King’s College Hospital became the major trauma centre for South East London. This prospective cohort study compares the incidence, changing demographic features and treatment outcomes of penetrating neck trauma in South East London over the previous 23 years.

**METHODS:**

Data were collected over a 12-month period (April 2010 to March 2011) and a selective management protocol was introduced to standardise initial investigations and further treatment.

**RESULTS:**

The past 23 years have seen a 550% increase in the incidence of penetrating neck injuries in South East London, with a marked increase in gun crime. Only 38% of cases underwent negative neck exploration in 2011 compared with 65% in 1987. Selective conservative management based on the absence of haemodynamic instability or radiological findings reduces length of hospital stay, lightens surgical workload and cuts costs without affecting morbidity or mortality.

**CONCLUSIONS:**

The increased incidence of penetrating neck injury is a reflection of more interpersonal violence rather than a consequence of the larger South East London trauma centre catchment area. Tackling this problem requires focus on wider issues of community prevention. Sharing of data between the four London trauma centres and the police is needed to help prevent interpersonal violence and develop a universal treatment algorithm for other institutions to follow.

Interpersonal violence, particularly as a result of gun or knife crime, has far reaching consequences for its victims and perpetrators, healthcare planning and society as a whole. The management of penetrating injuries to the head and neck is controversial with substantial variability in the treatment algorithms used throughout the world. In 1988 Ring’s College Hospital, London, published its experience of penetrating neck injuries (PNIs) where the otolaryngology team had managed 17 cases in the preceding 5 years.^1^

The last 20 years have seen the evolution of gang culture and associated violent assaults in inner city areas. There are 4,600 hospital admissions each year in England because of knife assaults.^2^ The police authorities of England and Wales recorded 277 fatal stabbings in 2008 with 86 in London alone.^3^ With the advent of the London trauma network in April 2010, Ring’s College Hospital became the major trauma centre for South East London while continuing to serve its traditional catchment area. This includes some of the most deprived and ethnically diverse boroughs in the UR.^4^

In this study we compare demographic features, treatment modality and outcomes of patients with penetrating injuries to the head and neck, managed from April 2010 to March 2011, with those published from the same institution two decades earlier. In doing so, we highlight the change in incidence and test a treatment protocol designed to standardise best practice in managing the growing burden of PNIs on healthcare and society.

## Methods

**Figure 1:**
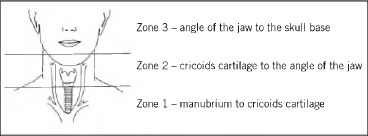
Anatomical boundaries of trauma neck zones as described by Monson *et al* in 1969^5^

The trauma team coordinator prospectively recorded demographic data, mechanism and extent of injury on all trauma patients with injuries of the head and neck that penetrated the platysma muscle. These data were examined over the 12-month period from 1 April 2010 to 31 March 2011. Patients were managed according to *Advanced Trauma Life Supporti* principles. The site of injury was described by neck trauma zones as set out by Monson *et al* in 1969 (Fig 1)^5^

Before 1 April 2010 there was no standardised protocol for initial investigation or further management. However, based on evidence gathered from literature and combining multidisciplinary experience, the following treatment was provided during the subsequent 12 months.

Patients who were difficult to assess due to central neurological deficit or who presented with hard signs of haemo-dynamic instability were transferred immediately to the operating theatre. These hard signs included:

- severe active bleeding
- large expanding haematoma
- unresponsive hypovolaemic shock (heart rate [HR] >90bpm, systolic blood pressure [BP] <100mmHg)
- diminishing radial pulse

Haemodynamically stable patients or those who presented with soft signs (haemoptysis, dysphagia, dysphonia, bubbling wound or peripheral nerve damage) underwent computed tomography (CT) angiography to rule out subclinical manifestations and were admitted for an observation period of 24 hours. Injuries that crossed the midline were subjected to contrast swallow to exclude damage to the aerodigestive tract.

Patients are presented to the multidisciplinary trauma team at the daily trauma ward round and only those with penetrating injuries sustained to the neck as defined by Monson *et al*^5^ were included in the study. These data have been supplemented by a medical records review to determine the investigations ordered, treatments dispensed and treatment outcomes including length of hospital stay (LOS). Postcode addresses of the injury location were obtained from the London ambulance service database. An operation involving only wound revision and closure in the operating room, without positive finding, was considered a non-ther-apeutic operation.

## Results

**Figure 2:**
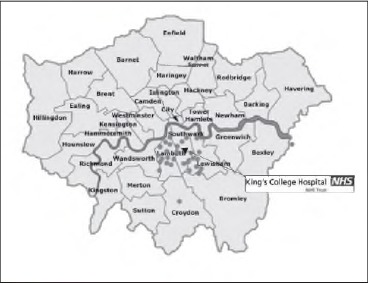
Map of London depicting the geographical distribution of penetrating neck trauma in South East London

During the 12-month study period, 31 patients with PNIs were treated, revealing an incidence of 4.3 per 100,000 population. Of the 31 patients, 29 were men and 2 were women, with a mean age of 30 years. An additional six patients sustained penetrating injuries to the face and skull but were not included in the dataset. The majority of injuries were sustained local to the hospital despite an increase in size of catchment area since gaining trauma centre status (Fig 2).

**Figure 3:**
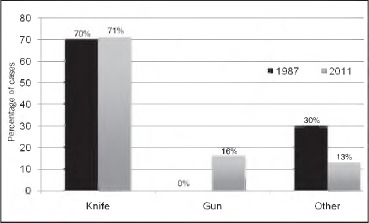
The change in mechanism of injury since Shotton’s original study^1^

Five patients sustained gunshot wounds and twenty-two sustained stab wounds. Four other cases included screwdriver, key, nail gun and snooker cue injuries. Overall, operative management was required more frequently with gunshot wounds (60%) than with stab wounds (19%). One patient died in the accident and emergency (A&E) department due to exsanguination. Figure 3 gives an overview of the mechanism of injury.

**Table 1:** Mode of investigation tabulated against zone of neck injuryw

Investigation	Zone 1	Zone 2	Zone 3	Total
Chest x-ray	1	2	0	**3**
Ultrasonography	1	1	2	**4**
Computed tomography angiography	6	7	9	**22**
Contrast swallow	2	1	0	**3**
Nil	2	0	3	**5**

Twenty-five patients (81%) were haemodynamically stable at the time of presentation whereas six (19%) were unstable. Twenty-two patients (71%) underwent CT angiography, of which two (9%) produced positive findings of internal jugular injury and pharyngeal shrapnel initiating surgical exploration. Five patients (16%) received no investigation and were managed conservatively without complications. Overall, there was no apparent correlation between neck zone and the modality of investigation chosen (Table 1).

**Figure 4:**
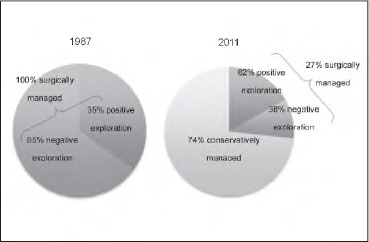
The change in management outcomes since Shotton’s original study^1^

Twenty-three cases (74%) were managed conservatively with a median LOS of 1 day. None of these required surgery at a later date and there were no mortalities. Eight patients (26%) were managed surgically with a median LOS of 4.5 days. Neck exploration revealed haematomas but no identifiable injuries in three (38%) of the patients who underwent surgery (Fig 4). Surgical management in the other five cases included repair of internal jugular vein, removal of shrapnel, ligation of the external carotid artery, repair of pharyngeal airway and endovascular coiling after several failed attempts of surgical haemostasis.

The presence of hard signs or investigative findings reliably predicted all those patients who required surgical intervention (sensitivity: 100%) whereas the absence of hard signs or investigative findings reliably excluded significant injuries requiring treatment in all cases managed conservatively (negative predictive value: 100%). Those selected for surgical exploration had injuries identified in the majority of cases (specificity: 88%).

## Discussion

From the time Ambroise Paré ligated both common carotid arteries and the jugular vein of a soldier with a traumatic neck injury in 1552, the initial assessment of a PNI has been one of the most controversial issues in trauma surgery. There remains no consensus view as to the perfect care pathway.

Mandatory exploration of all injuries violating the platysma reduced mortality successfully during the Second World War^6^ but 89% of operations found no injury to deep structures.^7^ In 1969 Monson *et al* divided the neck into three trauma zones to relate the anatomical structures at risk to management protocols (Fig 1).^5^ Angiography was recommended for injuries to zones 1 and 3 while mandatory exploration was still advocated for zone 2 due to the density of anatomical structures in it. A reduced negative exploration rate of 56% with no increase in morbidity or mortality was reported.^8^

Legerwood *et al* in 1980^9^ and Narrod and Moore in 1984^10^ showed that the absence of hard clinical signs of haemodynamic instability in penetrating limb injury accurately excluded arterial injuries needing surgical repair. Extrapolation of this finding into head and neck treatment algorithms was delayed because of concern that missed injuries could result in life threatening stroke.

The 1990s saw two studies^11^, ^12^ focus on physical examination alone to detect hard signs of vascular injury in neck zone 2 and the need for surgical repair. Clinical examination was shown to have an accuracy of greater than 99% in diagnosing such injuries with a false negative rate comparable with that of angiography.^12^ Clinical examination is quicker, less costly and involves fewer personnel. However, it is less likely to detect minimal injuries such as smooth narrowings, intimai irregularities, pseudoaneurysms and arteriovenous fistulas compared with angiography. The majority of such findings have no clinical significance.^13^ The additional cost and morbidity of routine angiography is therefore hard to justify.

Our experience suggests that the South East London trauma centre will see approximately 100 PNIs over the next 3 years, a dramatic increase of 550% on the number reported between 1984 and 1987 by Shotton.^1^ As depicted in Figure 2, the vast majority of injuries were sustained in the hospital’s original catchment area. Only a minority of patients were from the wider South East London trauma network. This suggests a true increase in incidence, which still remains low compared with trauma centres in North America^11^ and South Africa.^14^ Published algorithms from North America and South Africa reflect the healthcare resources of the country concerned, the socioeconomic circumstances of the local population and size of the geographical area served. Local management protocols therefore need to be resource and institution dependent.^15^

Our management protocol for PNIs is based entirely on haemodynamic status. There is no difference in initial management regarding the mechanism or site of injury. Decisions to treat conservatively are based on clinical signs. CT angiography is employed to further reduce the incidence of an undetected life threatening haemorrhage.

Prior to this study, initial investigation of patients presenting with a PNI to our unit varied. During the study period, five patients received no investigation at all. Our protocol standardises the management of PNIs at our institution and has been reinforced by a weekly educational meeting of the trauma team where the care pathways of patients who did not comply with protocol are revisited.

Although it is shown to be as sensitive and less costly than CT, Doppler ultrasonography is performed by the specialist vascular ultrasonography department and is not available after hours in our unit. When performed on patients in this study, subsequent CT angiography was used to confirm its findings, making its use superfluous.

Surgical intervention decreased from 100% in 1987 in Shotton’s report^1^ to 27% in the current series. Our protocol is far more specific than that adopted 27 years earlier. Neck exploration discovered no structural injury in 38% of the patients we managed surgically, compared with 65% in Shotton’s study (Fig 4). Had we applied the same policy of mandatory neck exploration, the incidence of negative exploration would have been 80%.

The median LOS for conservatively managed patients was 1 day as the protocol recommends a 24-hour observation period. The LOS of patients in this study may be exaggerated as our analysis does not account for the impact of other injuries sustained by the patients admitted with a PNI. For example, a chest x-ray performed for a zone 3 neck injury appears an unnecessary investigation unless we are aware of confounding penetrating chest injury. The few patients managed conservatively with prolonged LOS can therefore be explained by polytrauma requiring prolonged admission for treatment of other injuries and include those with a pneumothorax, peritonitis and spinal cord injuries.

We are aware that our study is limited by unknown pre-hospital mortality and patients who do not present to hospital or are managed in district general hospitals. Furthermore, it is not known how many subclinical vascular injuries are missed through a selective operative approach. Reassuringly, no patient required further treatment after discharge from hospital. Our work has encouraged the department to collect all relevant clinical details prospectively in accordance with the Cardiff model.^15^ With more comprehensive data collection, the true incidence of PNIs throughout the network may be even higher than stated currently.

**Figure 5:**
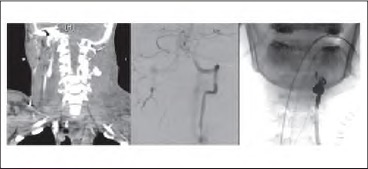
Computed tomography angiography and angiography demonstrating arteriovenous fistula of left vestibular artery and extensive endovascular coiling

Endovascular intervention was performed in one patient, an 18-year-old man who was stabbed on the left in zone 1 with a snooker cue. He presented to the A&E department at night with hypovolaemic shock (HR 138bpm, BP 84/50mmHg). Immediate neck exploration by a trauma surgeon failed to identify a bleeding point and the neck was closed. The wound drained 300ml in 30 minutes so the neck was re-explored by a multidisciplinary maxillofacial and vascular surgical team. Once again, no bleeding point could be identified. CT angiography revealed a vertebral artery arteriovenous fístula, which was successfully controlled after multiple attempts at endovascular coiling during normal working hours (Fig 5).

Given that injuries to the vertebral arteries are rare, the provision of a 24-hour on-call neuroendovascular service to offer a first line treatment at presentation for such patients is probably beyond the resources of our network. However, where possible, interventional radiology should be considered for injuries to zones 1 and 3, where bleeding can be very difficult to identify and control surgically.

While work optimising the management of patients with PNIs who reach hospital may decrease patient morbidity and mortality, prevention and wider issues of justice and safety are just as important. Only 23% of violent injuries treated in hospital are recorded by the police.^16^ The General Medical Council and the Department of Health released statements requesting that anonymised data should be forwarded of all wounds inflicted in a violent attack with a firearm, knife or any sharp object.^17^ The Cardiff model provided guidance on data that should be collected as well as methods to help overcome barriers to data collection.^15^

In 2012 the Association of Surgeons of Great Britain and Ireland (ASGBI) has been working with the police through the ‘Saving Lives, Reducing Violence’ initiative to improve data sharing between the two bodies.^18^ Data collection maps hotspots for violence. Local authorities are then able to link investment in police resources and provision of centres with the appropriate surgical skills to deal with these complex and unpredictable injuries in the areas where they are most prevalent.^19^

While local authorities are educating schoolchildren on the long-term health effects of knife injuries, the ASGBI and the Surgical Foundation are working together to roll out a damage control surgery course.^20^ Medical techniques for dealing with penetrating sharp injuries are very different from those for blunt injuries that make up the majority of trauma cases, and specific surgical training and protocols are needed to improve outcomes.

## Conclusions

We have witnessed a 550% increase in PNIs in South East London over the past 24 years. The increase in incidence is not a consequence of the widened catchment area resulting from centralisation of major trauma care to the South East London trauma centre at Ring’s College Hospital. Instead, it reflects the increase in interpersonal violence due to gun and knife crime between young men in the deprived inner city boroughs of South East London. This is also demonstrated in the national police statistics for violent crime in England and Wales.^3^

Based on our experience and data analysis, we have devised local guidelines for the South East London trauma centre at Ring’s College Hospital. These reflect local socioeconomic circumstances and healthcare resources to standardise the management of PNIs. We recommend that all injuries that penetrate the platysma presenting with soft signs undergo CT angiography and 24-hour observation. Injuries that cross the midline should undergo contrast swallow. Patients presenting with hard signs of haemodynamic instability should undergo emergency surgery. Interventional radiology should be considered early for zones 1 and 5, particularly when bleeding is refractory to surgical intervention.

NHS statistics suggest that the London trauma network saved 58 extra lives during its first year compared with the national average.^4^ The systematic collection and sharing of data between the four trauma centres of the London trauma network may allow further refinement of bespoke treatment algorithms that fit the needs and resources of other similarly placed institutions throughout the UR.
